# Genetic Diversity Analysis of Water Lily Germplasms Based on Morphological Traits and SSR Markers

**DOI:** 10.3390/plants14091365

**Published:** 2025-04-30

**Authors:** Min Wan, Wei Lu, Luxue Gao, Cuiping Li, Hanli Liu, Caibao Zhao, Xingmei Ai

**Affiliations:** College of Landscape Architecture and Horticulture, Southwest Forestry University, Kunming 650224, China; 18311611884@163.com (M.W.); 13388849422@163.com (W.L.); 13723844293@163.com (L.G.); 19912913706@163.com (C.L.); 18206747932@163.com (H.L.)

**Keywords:** tropical water lily, germplasms, morphological traits, SSR markers, genetic diversity

## Abstract

The study was conducted to identify and describe 34 morphological traits from 30 collected tropical water lily germplasms, including both viviparous and non-viviparous water lilies, along with a genetic diversity analysis utilizing 16 selected polymorphic SSR markers. The results revealed significant differences among various water lily germplasms. Specifically, the genetic diversity index for 15 qualitative traits ranged from 0.456 to 1.681, while the index for 19 quantitative traits exceeded 1.5, ranging from 1.532 to 2.024. and the coefficient of variation for these traits varied between 12.11% and 58.88%, indicating that the genetic diversity index of quantitative traits was significantly higher than that of qualitative characteristics. Seven principal components were extracted, accounting for 74.16% of the genetic information. By integrating the calculation of membership function values, germplasms with superior comprehensive characteristics were selected, including *N. ‘Purple Joy’*, *N. ‘Key Largo’*, and *N. ‘Eldorado’*. A total of 115 alleles were amplified using 16 pairs of SSR markers from the tested materials. The effective allele number (*Ne*) ranged from 2.01 to 8.02, Shannon’s information index (*I*) varied from 1.03 to 2.25, polymorphism information content (*PIC*) ranged from 0.57 to 0.90, and the genetic similarity coefficient was from 0.30 to 0.80. Based on the morphological traits and SSR molecular markers, the tested water lily germplasms were classified into four and five categories, respectively, showing certain similarities and differences. The morphological clustering effectively distinguished between viviparous and non-viviparous water lilies, while the SSR-based clustering did not show a significant correlation with viviparity. Principal component analysis indicated that different groups were relatively independent, while individuals within each group were concentrated. These results suggest that combining morphological traits and SSR analysis is an effective approach for evaluating the genetic diversity of water lilies and understanding the genetic relationships among germplasms and provide a valuable reference for utilizing viviparous germplasms and breeding new varieties.

## 1. Introduction

Water lily (*Nymphaea* L.) is a notable member of the family Nymphaeaceae and is highly valued for its role in ornamental and aquatic landscapes. It is distributed primarily in tropical, subtropical, and temperate regions [1,2]. Globally, there are over 50 species (including variants) of water lilies and more than 1000 horticultural germplasms. According to their ecotypes, water lilies are classified into five subgenera: *Nymphaea*, *Brachyceras*, *Lotos*, *Hydrocallis*, and *Anecphya* [3]. Notably, only the *Nymphaea* represents hardy water lilies, and the other four subgenera are all tropical species. Water lilies have multiple uses, including ornamental, medicinal, and edible purposes [4]. They are important raw materials for the food, pharmaceutical, and cosmetic industries [5,6,7], and play a crucial role in constructing water purification communities [8,9].

Water lilies have a long history of cultivation and exhibit broad ecological adaptability, showcasing rich germplasms with significant diversity in terms of flower and leaf traits, and although these morphological traits are easily influenced by genetic factors and environmental conditions, they can show the basic characteristics of germplasms more comprehensively and intuitively [10]. The earliest morphological classification system for the genus *Nymphaea* was established by Dr. Henry Conrad in 1905, breeders have made significant progress in identifying phenotypic traits in water lilies since the advent of numerical taxonomy. Studies have shown that floral organ structures [11], pollen size, outer wall shape, leaf shape [12], pollen color, and rhizome shape [13] could help effectively distinguish among different subgenera, determining the importance of morphological characteristics in the taxonomic classification of *Nymphaea*. By conducting analyses of diversity, correlation, principal components, and clustering, qualitative descriptions of phenotypic traits have been quantitatively assessed. Devi et al. [14] evaluated 40 morphological traits across 207 individuals from nine taxa of Indian water lilies and performed principal component analysis to identify three genetically distinct but morphologically identical taxonomic groups that explained 33%, 30%, and 12% of the total variance, respectively. Bai et al. [15] and Huang et al. [16] conducted a variance analysis, coefficient of variation analysis, and cluster analysis on the morphological traits of water lily germplasms, successfully classified these germplasms into different variety categories, and identified germplasms with high ornamental and horticultural value for landscape applications. Su et al. [17] and Pan et al. [18] performed comprehensive evaluations of morphological traits in introduced water lily germplasms, combining diversity analyses, correlation, principal components, and clustering, and transforming qualitative descriptions of morphological traits into quantitative evaluation. These evaluations revealed substantial variation in water lily morphological traits, with quantitative traits exhibiting greater diversity than qualitative traits.

Molecular markers are not dependent on morphological traits and the external environment, which could further reveal the genetic variation in germplasms at the DNA level, not only overcoming the inability of morphological/isozyme analyses to distinguish similar varieties but also understanding the genetic diversity of water lilies and the relationship between plant species more accurately. Currently, the genetic diversity analysis of water lilies primarily employs ISSR molecular markers [19], RAPD [13], and SSR molecular markers [20,21]. Mao et al. [22] amplified 207 polymorphic bands from 147 *Nymphaea* germplasms using 10 selected SRAP primers, achieving a 100% polymorphism rate. Qian et al. [23] identified 158 *Nymphaea* accessions using ITS, trnT-trnF, and rpl16 and conducted a comprehensive analysis combining sequence similarity, genetic distance analysis, and phylogenetic reconstruction, revealing that the combination of ITS with trnT-trnF (or rpl16) was more appropriate as a DNA barcode for water lilies. Dkhar et al. [13] used RAPD and ITS markers to speculate that the origin of some original species of water lilies may be the result of distant hybridization. Simple Sequence Repeat (SSR), known as microsatellite DNA [24], is currently recognized as one of the most important molecular markers due to its high polymorphism and robust stability [25]. As a heritable co-dominant marker, SSR can accurately identify the differences between germplasms and quickly identify varieties, effectively overcoming the limitations of traditional phenotypic analyses that can be influenced by environmental factors. SSRs have been extensively utilized in plant genetic breeding and studies of phylogenetic relationships [26,27]. Based on the above molecular marker technology, the genetic relationship of different water lily germplasms can be accurately and rapidly identified.

Morphological traits are influenced by environment and age, while molecular markers cannot directly reflect the differences in morphological traits. Most studies tend to focus on a singular approach to genetic diversity analysis (morphological or molecular marker). Therefore, the combination of these two approaches can yield complementary insights and provide a more accurate assessment of the genetic diversity within germplasms [28,29].

In practical production, tropical water lilies are typically propagated through seeds and bulbs. The *Brachyceras* subgenus within the *Nymphaea* genus represents a type of water lily distinguished by its unique aroma and trimerous stamens [30]. Some of its species or varieties exhibit viviparous traits at the leaf navel, where the growth of epiphyllous buds is primarily reliant on nutrients supplied by the leaves [31]. These viviparous seedlings have a low rate of virus infection, a high reproductive coefficient, and stable heritability [32], serving as a crucial supplementary method in water lily reproduction. Regarding environmental adaptation, this facultative reproductive trait in viviparous seedlings may enhance their ability to thrive in complex and variable natural environments. However, based on our previous research, despite the potential for production, the development of viviparous water lilies is limited by the decline or self-degradation of epiphyllous buds as the plants age [33]. Additionally, some farmers, being unaware, would discard the viviparous seedlings, which significantly impacts reproduction and population stability. In this study, we collected and preserved both viviparous and non-viviparous water lily germplasms. Although the quantity was limited, we observed significant morphological differences. However, the genetic diversity of these water lilies has not been thoroughly investigated. Therefore, it is essential to conduct genetic research to identify materials with superior characteristics. This research will provide valuable references for the protection and utilization of water lily germplasms as well as for genetic breeding. Such efforts are crucial for promoting innovation and enhancing the efficient use of germplasm resources.

## 2. Results

### 2.1. Analysis of Morphological Genetic Diversity in Tropical Water Lily Germplasms

#### 2.1.1. Differences in Plant Morphological Characteristics

**Figure 1:**
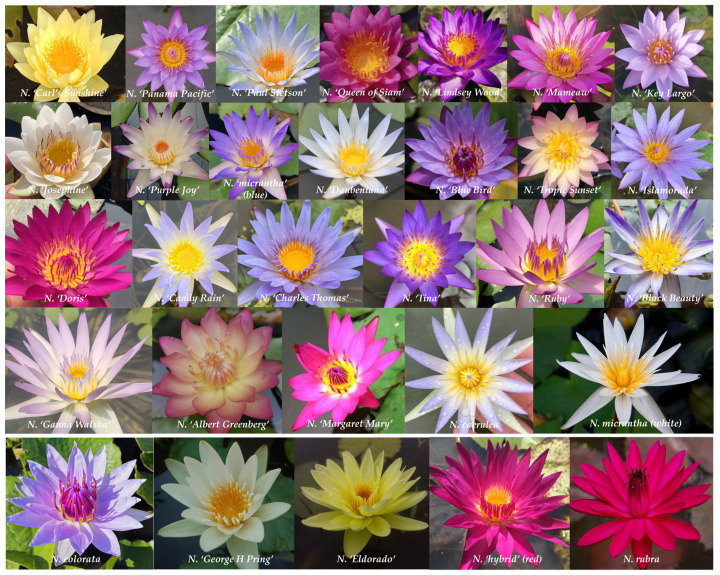
Flower organ traits of 30 water lily germplasms.

**Figure 2:**
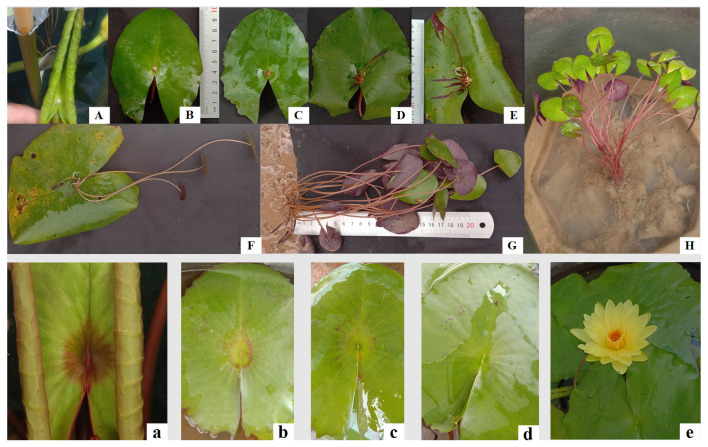
Morphological changes in viviparous and non-viviparous water lily leaves. Note: (**A**–**H**) represent viviparous water lily leaves; (**a**–**e**) represent non-viviparous water lily leaves.

The 30 water lily germplasms analyzed were all classified within the tropical water lily category. The flower colors of both viviparous and non-viviparous water lilies included white, amaranth, blue, violet, yellow, and chime color (Figure 1), indicating a high level of genetic diversity. The predominant flower forms were radial and cup-shaped, and the leaves exhibited typical serration; some were solid green without spots, while others displayed patches of varying sizes. Typical germplasms include *N. ‘Carl’s Sunshine’*, *N. ‘Lindsey Wood’*, *N. ‘Doris’*, and *N. ‘Ganna Walska’*, all of which significantly enhance the ornamental value of the leaves. The most distinctive morphological contrast between viviparous and non-viviparous leaves is observed at the leaf navel (Figure 2). During the submerged leaf curling stage, viviparous leaves develop a protrusion with white pubescence attached to the leaf navel during the submerged leaf curling stage (Figure 2A). As these leaves expand, epiphyllous buds gradually grow into viviparous seedlings (Figure 2B–F). Following leaf senescence characterized by yellowing and withering, these viviparous seedlings can rapidly emerge from the mother plant (Figure 2G and H). In contrast, non-viviparous leaves exhibit no morphological changes at the leaf navel throughout their development, from curled juvenile stages to mature senescence. While the leaf navel remains smooth and unchanged during this process, surrounding tissues undergo dynamic color shifts as they expand before ultimately experiencing progressive chlorosis.

#### 2.1.2. Genetic Diversity Analysis of Qualitative Traits

A total of 15 qualitative traits were evaluated across 30 viviparous and non-viviparous water lily germplasms, with a systematic analysis conducted on the frequency distribution and diversity index of each trait (Table 1). The genetic diversity index for these qualitative traits ranged from 0.456 to 1.681. Among them, young leaf color, leaf form, petal color, flower fragrance, petal shape, and anther color all exhibited diversity indices greater than 1.0. Notably, the highest genetic diversity index was observed in petal color at a score of 1.681, with violet flowers showing the highest distribution frequency at 33.33%. This was followed by leaf form with a diversity index of 1.366 and anther color with an index of 1.346. Specifically, rounded and elliptic leaves represented 30.00% of their respective traits, while purple anthers accounted for 46.67%. In contrast, the diversity index of viviparity of leaf was the lowest at 0.456. Regarding floral characteristics, flower fragrance predominantly exhibited slight intensity, with a diversity index of 1.062. However, certain germplasms, such as *N. ‘Eldorado’* and *N. ‘hybrid’* (red), displayed strong fragrances that render them particularly suitable for use in flower tea beverages. Petal shape was predominantly presented as lance-shaped, constituting approximately 46.67% of observations and possessing a diversity index of 1.072. The inflorescence shape predominantly appeared cup-shaped among observed germplasms at a rate of about 60.00%. For sepal color, distinct differences were noted, with 80.00% of varieties exhibiting lighter inner colors contrasted by darker outer pigmentation along with diverse spot patterns on the sepals, resulting in a diversity index of 0.957. Analysis concerning leaf traits revealed that separated leaf auricles comprised 53.33% of specimens examined. Most germplasms exhibited both young and mature leaves, mainly characterized by green hues often accompanied by irregular brown spots. This spotted characteristic was observed in 40.00% of the collected germplasms, yielding a diversity index of 0.950. Furthermore, fruit shapes varied significantly among the different germplasms, with pineapple-shaped fruits accounting for 13.33%, apple-shaped fruits for 53.33%, and peach-shaped fruits for 33.33%; this resulted in an overall diversity index of 0.969. These findings underscore a rich genetic diversity in both organ-specific traits and leaf characteristics among the evaluated water lily germplasms.

#### 2.1.3. Variant Analysis of the Quantitative Traits

The 19 quantitative traits of viviparous and non-viviparous water lilies showed significant variation (Table 2). All genetic diversity indices were greater than 1.5, ranging from 1.532 to 2.024. Notably, traits such as petiole diameter, and flower density displayed exceptionally high diversity indices (*H’* > 2.0), and the remaining quantitative traits mostly had diversity indices between 1.8 and 1.9. The analysis revealed that the coefficients of variation (CV) for 19 quantitative traits ranged from 12.11% to 58.88%, with an average of 25.12%. Among these traits, the carpel number exhibited the smallest coefficient of variation at 12.11%. In contrast, the diameter of epiphyllous buds had the largest coefficient of variation at 58.88%, followed by the number of epiphyllous buds at 56.56% and flower height at 33.84%. This indicates that these three quantitative traits displayed substantial variation within the test materials and possess significant potential for genetic improvement.

### 2.2. Comprehensive Evaluation of Morphological Traits of Water Lily Germplasms

#### 2.2.1. Principal Component of Morphological Traits

The original dataset consisted of 34 morphological traits derived from viviparous and non-viviparous water lily germplasms. These data were standardized using the range standardization method and subsequently analyzed through principal component analysis (PCA) (Table 3). Seven principal components were extracted, each exhibiting eigenvalues greater than 1.5, collectively accounting for 74.157% of the total variance. This suggests that these components effectively capture the morphological characteristics of different water lily germplasms. The first principal component (PC1) had an eigenvalue of 9.501, representing 27.943% of the total variance. The morphological traits with the highest absolute eigenvector values in this component were leaf width and leaf length, indicating that PC1 primarily reflects variations in leaf morphology. The PC2 had an eigenvalue of 4.238, accounting for 12.465% of the variance. Within this component, the diameter of epiphyllous buds exhibited the highest eigenvector value, followed by viviparity of leaf. These results demonstrate that the PC2 primarily represents a synthesis of viviparity-related traits. The PC3 had an eigenvalue of 3.979 and accounted for 11.703% of the variance. Within this component, leaf color exhibited the highest absolute value for its eigenvector, followed by leaf spot and young leaf color, indicating that it represents variations in leaf color characteristics across different developmental stages. The cumulative contribution rate of the first three principal components reached 52.111%, all linked to leaf morphological characteristics. The PC4 had an eigenvalue of 2.350 and contributed 6.911%, primarily associated with sepal color and flower density. The PC5, with an eigenvalue of 1.829, accounted for an additional contribution rate of 5.380%, mainly related to fruit shape and sepal shape. The PC6 had an eigenvalue of 1.751, accounting for 5.151% of the total variance, and was primarily related to flower fragrance and petal shape. The PC7 demonstrated an eigenvalue of 1.565, contributing 4.603%, with another color demonstrating the highest absolute eigenvector value. The cumulative contribution rate of the last four principal components was 22.046%, predominantly encapsulating morphological variation in floral organs. The findings indicated that key morphological traits such as leaf length, leaf width, leaf color, young leaf color, leaf spots, viviparity of leaf, sepal color, fruit shape, sepal shape, flower fragrance, petal shape, anther color, flower density, and the diameter of epiphyllous buds are essential for differentiating between viviparous and non-viviparous water lily germplasms.

#### 2.2.2. Correlation Analysis of Morphological Traits

**Figure 3:**
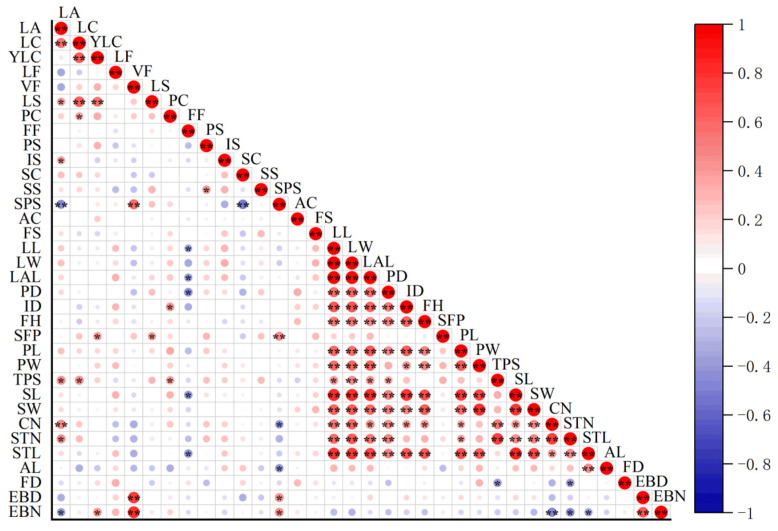
Correlation analysis between 34 morphological traits of water lily germplasms. Note: LA, LC, YLC, LF, VF, LS, PC, FF, PS, IS, SC, SS, SPS, AC, FS, LL, LW, LAL, PD, ID, FH, SFP, PL, PW, TPS, SL, SW, CN, STN, STL, AL, FD, EBD, and EBN are leaf auricle, leaf color, young leaf color, leaf form, viviparity of leaf, leaf spot, petal color, flower fragrance, petal shape, inflorescence shape, sepal color, sepal shape, sepal and petal spot, anther color, fruit shape, leaf length, leaf width, leaf auricle length, petiole diameter, inflorescence diameter, flower height, single flowering period, petal length, petal width, total number of petals, sepal length, sepal width, number of carpels, number of stamens, stamen length, anther length, flower density, diameter of epiphyllous buds, and number of epiphyllous buds. * indicates significant correlation at 0.05 level; ** indicates significant correlation at 0.01 level. Red represents positive correlation; blue represents negative correlation. The darker the color, the stronger the correlation.

A correlation analysis was conducted on 34 morphological traits of both viviparous and non-viviparous water lily germplasms (Figure 3). The findings revealed varying degrees of correlations among the different morphological characteristics. Among the 15 qualitative traits, 11 pairs demonstrated highly significant correlations (*p* < 0.01). Extremely significant positive correlations (*p* < 0.01) were identified between leaf auricle and leaf color, as well as carpel number; between mature leaf color and juvenile leaf color along with leaf spots; and between leaf viviparity and several other traits, including sepals, petal spots, diameter of epiphyllous buds, and number of epiphyllous buds. Conversely, a significant negative correlation (*p* < 0.01) was found between sepal and petal spots concerning leaf auricle and sepal color. This suggests that variations in leaf and floral organ characteristics may influence the patterns of leaf and flower spots. Among the 19 quantitative traits, five traits, including single flowering period, anther length, flower density, diameter of epiphyllous buds, and number of epiphyllous buds, showed no significant differences from other traits (*p* > 0.05). In comparison, the remaining 14 quantitative traits showed significant or extremely significant differences. Notably, there was a highly significant positive correlation among leaf length and width and leaf auricle length (*p* < 0.01). Additionally, several floral organ characteristics—including petal length, petal width, total number of petals, sepal length, sepal width, number of carpels, total number of stamens, and stamen length—also demonstrated generally significant or highly significant positive correlations. This indicates a positive association between leaf growth and floral organ development. For future breeding programs, priority should be given to selecting large-flowered and broad-leaved germplasms that exhibit favorable ornamental qualities as parental lines for cultivation. Furthermore, for parental lines aimed at hybridization purposes, germplasms that show better coordination in terms of petal length-to-width ratios and numbers of female and male reproductive organs should be chosen for propagation.

#### 2.2.3. Comprehensive Evaluation of Water Lily Germplasms

After standardizing the 34 morphological traits of the tested materials, we substituted the values into the factor score coefficients corresponding to the seven principal components. The functional expressions for principal component scores are defined as follows: F_1_ = 0.117x_1_ + 0.016x_2_ + 0.003x_3_ + … − 0.135x_34_, with similar equations applicable to F_2_ through F_7_. Subsequently, the principal component value (*F*) was derived using the formula: F = (0.2794F_1_ + 0.1247F_2_ + 0.1170F_3_ + 0.0691F_4_ + 0.0538F_5_ + 0.0515F_6_ + 0.0460F_7_)/0.7416, where the coefficients represent the variance contribution rates of each principal component as weighting factors. Finally, the germplasms were evaluated and ranked based on their *F*-values (Table 4), with higher values indicating superior overall morphological performance. Seven cultivars achieved comprehensive scores exceeding 1.0. Among these, *N. ‘Purple Joey’* demonstrated exceptional overall traits within the viviparous category, featuring compound flower coloration and distinctive spots on both sepals and petals. It was closely followed by *N. ‘Key Largo’*, notable for its blue-purple flowers, abundant leaf patches, and high ornamental value. Meanwhile, *N. ‘Eldorado’* was recognized as the top performer among non-viviparous germplasms, characterized by its large leaves, yellow petals, and anthers, along with prolific flowering—making it a key raw material for scented tea production.

#### 2.2.4. Cluster Analysis of Water Lily Germplasms Based on Morphological Traits

**Figure 4:**
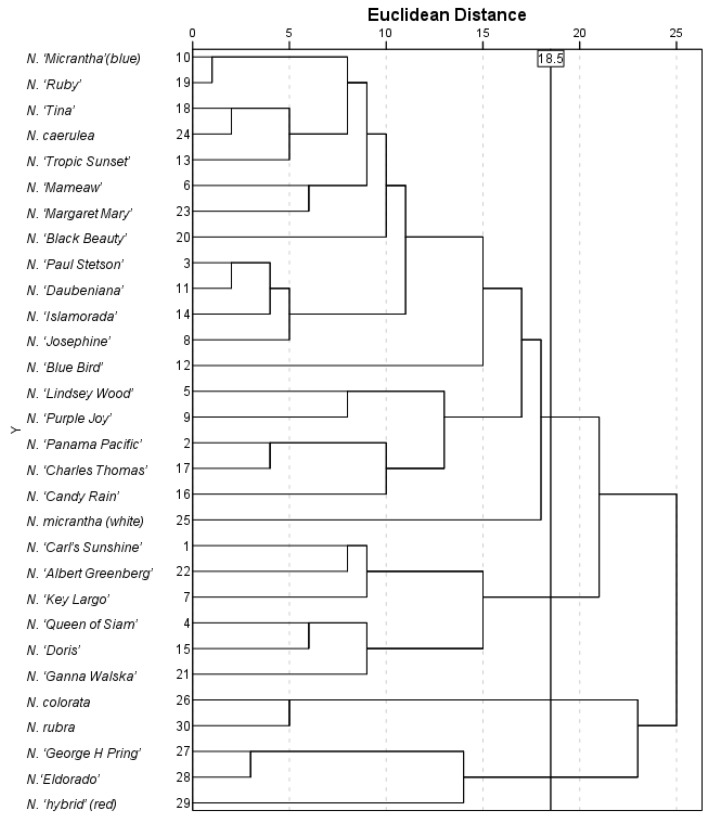
Cluster analysis of 30 water lily germplasms based on morphological traits.

Cluster analysis that incorporates all morphological traits may lead to interference among water lilies with heterogeneous characteristics. To address this, 14 key morphological traits were selected through principal component analysis based on their contribution rates. These traits were subsequently employed for hierarchical clustering of the germplasms (Figure 4). The results demonstrated that the 30 tested water lilies were classified into four distinct clusters at a Euclidean distance of 18.5. Cluster I comprised 19 viviparous water lily germplasms, including *N. ‘Purple Joey’*. The primary morphological characteristics of this group included uniformly green leaves without spots, predominantly separated leaf auricles, varied anther coloration, and relatively large viviparous bud diameters (with over 72% of germplasms exceeding 0.50 cm), as well as bud quantities ranging from 1.5 to 4.5. Notably, *N. micrantha* showed close phylogenetic relationships with multiple viviparous germplasms within this cluster. Cluster II comprised six viviparous germplasms, including *N. ‘Key Largo’*, *N. ‘Carl’s Sunshine’*, *N. ‘Queen of Siam’*, *N. ‘Doris’*, *N. ‘Ganna Walska’*, and *N. ‘Albert Greenberg’*. Key morphological traits of this cluster featured mature leaves displaying green or brown-green variegation, abundant leaf spots, orbicular to ovate leaf shapes, a consistently delicate floral fragrance, distinct coloration between inner and outer sepals, smaller viviparous bud diameters (ranging from 0.28 to 0.67 cm), and bud quantities ranging from 2.3 to 3.0. Cluster III comprised *N. colorata* and *N. rubra*, while Cluster IV included *N. ‘George H. Pring’*, *N. ‘Eldorado’*, and *N. ‘Hybrid’* (red), totaling five non-viviparous water lilies. Shared characteristics within both clusters included uniformly green color in both juvenile and mature leaves (generally exhibiting minimal or no spotting), as well as the absence of spots on sepals and petals. Distinctively, resources in Cluster IV exhibited overlapping leaf auricles and a pronounced floral fragrance. The clustering results effectively segregated viviparous and non-viviparous water lilies. However, morphological clustering analysis can only differentiate germplasms with pronounced morphological divergence and identify biological clustering patterns; it does not elucidate the genetic relationships among the germplasms.

### 2.3. SSR Genetic Diversity Analysis of Water Lily Germplasms

#### 2.3.1. SSR Polymorphism Analysis

Utilizing 16 pairs of screened SSR primers, a total of 115 allelic loci were amplified in the tested water lily materials, yielding an average of 7.19 alleles per locus (Table 5). The number of alleles (*Na*) detected by different primers ranged from 4 to 10; among them, primers NtG014, NtG115, and NtG188 exhibited the highest number of loci, each showing a count of 10. The effective number of alleles (*Ne*) varied from 2.01 (NtG011 and NtG107) to 8.02 (NtG188), with an overall average of 5.56. The average Nei’s diversity index (*H*) was 0.33, with the highest being 0.47 and the lowest 0.20. Shannon’s diversity index ranged from 1.03 to 2.25 and primer NtG188 exhibited the highest value, while NtG011 recorded the lowest, giving an average of 1.65. The observed heterozygosity (*Ho*) and expected heterozygosity (*He*) ranged from 0.05 (NtG006) to 0.43 (NtG152) and 0.45 (NtG006) to 0.95 (NtG146), respectively, with averages of 0.25 and 0.68. It is noteworthy that *Ho* values for all loci were lower than their corresponding *He* values. The polymorphism information content varied between values of 0.57 and up to a maximum of 0.90, averaging approximately 0.78 across all primers tested. All primers demonstrated *PIC* values exceeding the threshold of 0.50, indicating that these SSR primers demonstrate strong polymorphism in the tested water lily materials as well as reflecting relatively high genetic diversity within the population.

#### 2.3.2. Genetic Similarity Analysis

Using the NTSYS-PC 2.10e software, we calculated the genetic similarity coefficients between viviparous and non-viviparous water lilies. The coefficients ranged from 0.30 to 0.80, with an average of 0.57. The genetic similarity coefficient between *N. ‘Panama Pacific’* and *N. ‘Key Largo’* was the lowest (0.30), suggesting that they exhibit the most significant genetic variance and the most distant genetic relationship. In contrast, *N. ‘Blue Bird’* and *N. ‘Albert Greenberg’* showed a higher genetic similarity coefficient of 0.80, suggesting a closer genetic relationship.

#### 2.3.3. Clustering Analysis of Water Lily Germplasms Based on SSR Markers

**Figure 5:**
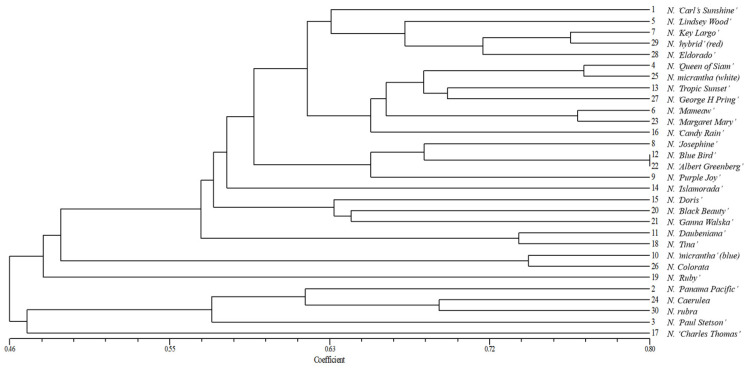
UPGMA tree map of 30 water lily germplasms based on SSR markers.

The clustering results were converted into co-representation matrices, and Mantel’s test was conducted to compare these matrices with the similarity coefficient matrices. The results showed that the correlation coefficient for morphological traits was 0.679, while the correlation for molecular markers was stronger at 0.762. Both values indicated highly significant correlations, demonstrating that the clustering patterns from both genetic diversity analyses reliably reflect the genetic relationships among germplasms. Furthermore, we analyzed the correlation between the similarity matrices derived from morphological and molecular data; a low correlation (r = 0.20) was observed, suggesting limited congruence between morphological and genetic diversity patterns. UPGMA cluster analysis revealed that 16 pairs of SSR primers effectively differentiated the tested materials, given the morphological cluster analysis, categorizing the water lilies into five clusters with a genetic similarity coefficient threshold at 0.50 (Figure 5). Category I included germplasms that ranked among the top ten in morphological evaluation, with the exception of *N. ‘Panama Pacific’*, alongside non-viviparous water lilies such as *N. ‘George H Pring’* and *N. ‘hybrid’ (red)*. Among these, *N. ‘Blue Bird’* and *N. ‘Albert Greenberg’* exhibited the closest genetic relationship. Category II comprised two germplasms, *N. ‘Micrantha’ (blue)* and *N. colorata*; the viviparous water lilies, *specifically N. ‘Ruby’* (category III) and *N. ‘Charles Thomas’* (category V) were each assigned to separate groups. Category IV contained four water lily accessions: *N. ‘Panama Pacific’*, *N. caerulea*, *N. ‘Paul Stetson’* (a viviparous germplasm), and *N. rubra* (a non-viviparous germplasm). The UPGMA dendrogram illustrated that viviparous and non-viviparous water lily germplasms were not distinctly separated, indicating that the clustering results did not show a significant correlation with viviparity. Among these, the non-viviparous water lilies *N. ‘George H Pring’*, *N. ‘Eldorado’*, and *N. ‘hybrid’* (red) exhibited relatively close genetic relationships while demonstrating more distant relationships with *N. colorata* and *N. rubra*.

#### 2.3.4. Principal Component Analysis of Water Lily Germplasms Based on SSR Markers

**Figure 6:**
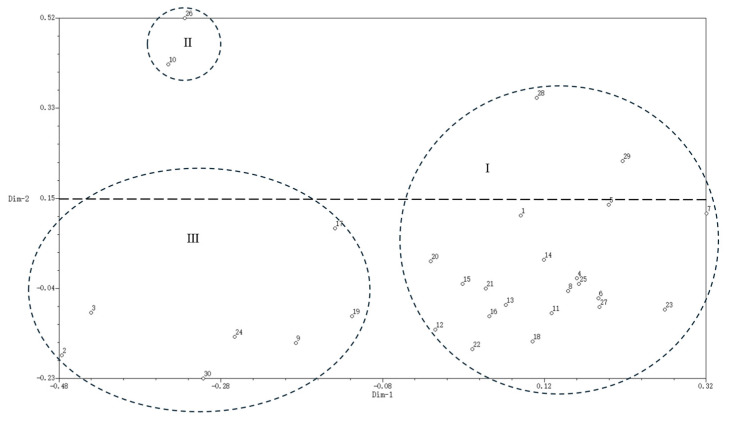
Two-dimensional scatter plot of principal components analysis of 30 water lily germplasms. Note: (1–30) correspond to Table 6.

A principal component analysis (PCA) was conducted on 30 water lily germplasms using a genetic similarity coefficient matrix derived from SSR molecular markers, facilitated by NTSYS-PC 2.10e software (Figure 6). The analysis revealed that germplasms located near each other indicate a strong genetic relationship, while those further apart suggest a more distant genetic association. The first principal coordinate accounted for 12.84% of the variance among the germplasms, and the second principal coordinate explained 8.93%. The viviparous and non-viviparous water lilies were categorized into three groups based on their positions in Dim-1 and Dim-2 principal coordinates. Group I consisted of 21 water lilies, primarily sourced from Hainan Province, including *N. ‘Blue bird’* and *N. ‘Albert Greenberg’*, which exhibited the highest genetic similarity coefficient. Conversely, the non-viviparous water lilies *N. ‘Eldorado’* and *N. ‘hybrid’ (red)* originating from Guangxi Province were clustered significantly farther away from other materials. In comparison with clustering analysis, all materials except for *N. ‘Purple Joy’* fell within the first category, indicating that those in the same cluster also displayed a relatively concentrated distribution trend in PCA. Group II comprised two germplasms, *N. ‘Micrantha’ (blue)* and *N. colorata*, both from Nanjing—which were distinctly positioned within the second category of clustering analysis, suggesting a close genetic relationship between them, although they are genetically distant from each other. Group III included seven water lilies distributed across Categories III, IV, and V according to the cluster analysis; most of these originated from Nanjing. In conclusion, the PCA performed on the water lily germplasms based on SSR markers aligns closely with the findings from the clustering analysis and demonstrates a significant correlation with their geographical origins.

## 3. Discussion

### 3.1. Evaluation of Viviparous and Non-Viviparous Water Lily Germplasms Based on Morphological Traits

The current classification of subgenera and interspecies within water lily germplasms remains inadequately defined, as it predominantly relies on traditional taxonomy to ascertain phylogenetic relationships among species. The richness and variability of morphological characteristics in water lilies are the prerequisites for the evaluation and utilization of these germplasms, reflecting the complex interaction between genetic factors and environmental influences. This study analyzed 34 morphological traits, revealing that six qualitative traits demonstrated genetic diversity indices exceeding 1.0, with flower color demonstrating the highest diversity index at 1.681. This trait was identified as a critical determinant in assessing ornamental value. This trend likely reflects the selective breeding practices aimed at enhancing ornamental characteristics within germplasms. Additionally, both anther color and leaf morphology emerged as significant factors in ornamental evaluation. Zhang et al. [34] demonstrated that the coefficient of variation was greatest for anther color, followed by flower color. This finding partially diverges from our results, which may be attributed to variations in the germplasms utilized across studies. All quantitative traits analyzed in this study exhibited genetic diversity indices above 1.5, indicating substantial morphological variation among water lilies; notably, higher diversity was observed in quantitative traits compared to qualitative ones. The phenotypic coefficient of variation offers insights into the inherent characteristics of varieties as well as differences among individual plants. Moreover, this coefficient can indicate the degree of dispersion for quantitative traits [35]; a larger coefficient suggests greater dispersion of morphological traits alongside a more diverse genetic background. Among the limited water lilies tested in this study, 90% of quantitative traits across various water lily germplasms exhibited coefficients of variation exceeding 15%, indicating significant differences among the tested varieties. Greater genetic diversity is associated with enhanced adaptability to stressful environments. This may be linked to the facultative reproductive characteristics of water lilies, which are capable of both asexual and sexual reproduction, resulting in complex genetic relationships. There are still some unexploited viviparous resources that may have rich genetic diversity and possess potential application value for the quality improvement of water lilies.

Principal component analysis (PCA) serves as a dimensionality reduction technique that relies on inter-indicator correlations to transform multiple morphological traits into principal components through orthogonal transformation. This methodology facilitates the simplification of evaluation indices by utilizing eigenvector values as weighting coefficients to construct composite evaluation functions. PCA has been widely adopted in the assessment of germplasms across various species, including woody plants like apple [36] and pear [37], as well as herbaceous plants such as *Hemerocallis* [29], maize [38], and cardamom [39], successfully screening out germplasm with excellent traits. This plays a significant role in the innovation of germplasm and the genetic improvement and utilization of varieties. In this study, 34 morphological traits were condensed into seven independent principal components, achieving a cumulative contribution rate of 74.157%. 14 key eigenvectors associated with leaf and floral organ morphology were identified, representing a substantial portion of the information regarding the morphological traits of the tested materials. It is evident that a few critical trait modifications may lead to variations in the morphological characteristics of water lilies. Through overall evaluation of *F*-values across germplasms, superior germplasms demonstrating exceptional composite traits—including *N. ‘Purple Joy’*, *N. ‘Key Largo’*, and *N. ‘Eldorado’*—were identified. These selected germplasms show significant potential for advancing both viviparous and non-viviparous water lily innovation. The analysis of correlations between morphological traits can reveal associations among traits. A study investigating the correlation of morphological traits in water lily germplasms revealed that the viviparity of leaves exhibited significant positive correlations with sepal and petal spots, the diameter of epiphyllous buds, and the number of epiphyllous buds (*p* < 0.01). Conversely, sepal and petal spots demonstrated an extremely significant negative correlation with leaf auricle and sepal color (*p* < 0.01). These findings indicated that the morphological traits of water lilies are interconnected, with most quantitative traits related to leaf and floral organ morphology exhibiting pronounced or highly significant positive correlations. The results suggest that morphological traits in water lilies interact synergistically; quantitative characteristics linked to leaves and floral organs predominantly exhibit strong or extremely strong positive correlations. Variations in a select few key traits may lead to marked differences in the overall morphological characteristics of water lilies. This implies that observed correlations reflect differentiated yet coordinated growth patterns among various organs during water lily development [40]. Furthermore, through morphological cluster analysis, Su et al. [17] could separate viviparous and non-viviparous water lilies clearly, which is similar to the results of this study.

The limited number of viviparous water lilies collected in this study may have introduced some bias in the experimental results and cannot provide a more comprehensive view of the genetic diversity. *N. micrantha* is a high-quality initial species and has been used as a parent to breed several viviparous water lilies, including *N. ‘Blue Brid’*, *N. ‘Charles Thomas’*, and *N.* ‘Daubenina’, etc. Morphological analysis indicates that these germplasms cluster together, reflecting their strong genetic relationships. However, in addition to the aforementioned germplasms, certain water lilies can propagate through floral viviparity; for example, hardy water lilies such as *N. ‘Colonel A.J. Welch’*, *N. ‘Cherokee’*, *N. ‘Perrys Cactus Pink’*, *N. ‘Perrys Pink Delight’*, and *N. ‘Perrys Viviparous Pink’* exhibit this reproductive trait. Among tropical water lilies, the night-blooming *N. ‘Prolifera’* produces multiple co-flowering buds from a single blossom, while the toothed water lily *N. ‘Lotus’* occasionally displays floral viviparity. Beyond their facultative reproductive reproduction characteristics, these water lilies are also highly valued for their ornamental appeal. We conducted a preliminary analysis of the genetic diversity present in limited germplasms of viviparous water lilies. By utilizing these unique resources, which exhibit distinct phenomena of leaf navel and flower viviparity, we aim to establish them as parental lines for inter-subgeneric hybridization breeding to cultivate superior varieties. Furthermore, we will continue our extensive collection efforts of viviparous water lilies to enhance their genetic diversity evolution, thereby further promoting the conservation, development, and utilization of this remarkable group of plants.

### 3.2. Genetic Diversity Analysis of Water Lily Germplasms Based on SSR Molecular Markers

SSR molecular markers are characterized by their abundance, high stability, and polymorphism, rendering them valuable for genetic diversity analysis of germplasms. The water lily, characterized by its relatively small genome and basal position within the angiosperm phylogenetic tree, represents a crucial lineage for investigating the origin and evolution of flowering plants [41,42]. Mao et al. [20] employed Microsatellite Identification (MISA) software to identify SSR loci in the chromosomal genome sequences of *Nymphaea*. From a total of 150 candidate SSR primer pairs, 11 were selected to amplify 307 polymorphic alleles across 147 *Nymphaea* germplasms, and the genetic diversity parameters recorded included the number of alleles (*Na*) = 5.36, the effective number of alleles (*Ne*) = 1.072, the genetic diversity index (*H*) = 0.056, Shannon’s information index (*I*) = 0.114, and polymorphism information content (*PIC*) = 0.53. In a subsequent study, Su et al. [43] employed 16 SSR primer pairs to detect a total of 205 alleles across 240 *Nymphaea* germplasms. Our research results of SSR analysis showed that *Na*, *Ne*, *H*, *I*, *Ho*, *He*, and *PIC* for water lily germplasms were 7.19, 5.56, 0.33, 1.65, 0.25, 0.68, and 0.78, respectively; these mean values significantly exceeded those reported by Mao et al. [20]. In this study, most viviparous germplasms exhibited high heterozygosity due to their hybrid progeny status, while the set of 16 SSR primer pairs demonstrated considerable polymorphism with an average effective allele number accounting for 77.33% of the total alleles analyzed. Notably, the primer sequences of NtG006, NtG014, NtG051, NtG115, NtG152, NtG156, and NtG188 were classified as highly polymorphic (*PIC* > 0.8). These results indicate the efficiency of the selected SSR markers for evaluating genetic diversity in *Nymphaea*.

### 3.3. Cluster Analysis of Water Lily Germplasms Based on Morphological Traits and SSR Molecular Markers

Morphological traits and molecular markers, when analyzed independently, often reflect distinct evolutionary selective pressures and show relative independence [44]. Consequently, the integration of these two types of data facilitates a comprehensive understanding of observable morphological characteristics alongside the underlying genetic divergence [45]. Plant morphological diversity is the performance of the combined effects of genetic and environmental factors. Under the condition of maintaining environmental consistency as much as possible, we stabilized the morphological characteristics of water lily plants and gained an overall understanding of the diversity level of the collected viviparous water lily germplasms. This understanding serves as a crucial prerequisite for the identification, evaluation, protection, and utilization of germplasms and plays a significant role in determining the adaptability, trait potential, genetic research, and breeding of germplasm resources.

In this study, water lily germplasms were classified into four major categories based on their morphological characteristics. Cluster analysis effectively distinguished between viviparous and non-viviparous water lilies, grouping materials with similar leaf and flower organ characteristics together, and each individual retained its unique characteristics. Conversely, the SSR marker cluster analysis categorized the tested materials into five groups. *N. ‘Charles Thomas’* and *N. ‘Ruby’* were each placed in separate categories, while the viviparous and non-viviparous germplasms were dispersed across the remaining three major groups. The results from the clustering analyses indicated no clear morphological classification characteristics. A comparison of the clustering outcomes from both methods reveals that the morphological traits and SSR markers do not fully align; there is only a partial agreement. This observation aligns with findings reported by Chalbi et al. [28] and Manco et al. [46]. This phenomenon may stem from the fact that morphological traits are more influenced by artificial selection during the breeding processes and may be subject to measurement errors. In contrast, SSR markers exhibit greater conservation and are less affected by artificial breeding or selection, but can be influenced by environmental conditions and dominant-recessive gene interactions, resulting in unstable genetic expression [47].

Mantel’s test revealed that both morphological and molecular marker-based clustering effectively reflected genetic relationships among germplasms, with correlation coefficients of 0.679 and 0.762. However, the weak correlation between morphological and molecular data indicates that there is no significant relationship (r = 0.20). This might be related to the insufficient sample in this study that reduced the statistical power. Nevertheless, these two methods complement each other. Maccaferri et al. [48] and Petrović et al. [49] also reported a weak correlation between morphological and molecular data, which might be caused by the environmental influences or unreliable pedigree data. In contrast, SSR molecular markers can provide deeper insights into the genetic similarities and morphological relationships among different lotus varieties at the molecular level. Some varieties may exhibit low morphological differentiation, but precise identification can still be achieved through molecular marker clustering analysis [50,51]. It is essential to recognize that molecular marker analysis cannot completely replace morphology-based analytical methods, as both have unique advantages and can complement one another in various identification tasks. Therefore, integrating morphological traits with molecular markers for a comprehensive analysis significantly enhances the accuracy and reliability of identifying lotus varieties [45].

This research demonstrates that both morphological and molecular analyses of the viviparous water lily have resulted in distinct clustering patterns. Resources exhibiting consistent morphological characteristics are grouped together, while genetic diversity indices, such as *He* and *PIC*, reveal significant variations among these germplasms. Notably, when conducting cluster analysis on individuals with close genetic relationships, the results do not strictly adhere to geographical origins, suggesting that there is no inherent correlation between genetic distance and the source of the germplasms. Both methodologies have proven invaluable for evaluating water lily germplasms, enabling the selection of specific resources from various groups based on targeted requirements. Currently, breeding strategies for water lilies primarily rely on traditional hybridization or morphological selection that focuses on floral organ traits. This approach often results in a homogenization of varieties and may lead to the marginalization or underutilization of valuable germplasms. Such selective pressure can cause a loss of unique genetic material and potentially endanger rare lineages with specialized adaptability. To address these challenges, we plan to expand the collection scope and quantity of viviparous water lilies, enhance the exchange and sharing of germplasm materials, and integrate germplasm resource science with genomics to uncover excellent and unique gene resources. This will provide a favorable foundation for the protection, utilization, innovation, and new variety breeding of viviparous water lily germplasms.

## 4. Materials and Methods

### 4.1. Experimental Material

For the morphological characterization in situ, viviparous seedlings of 30 water lily germplasms were collected from four provinces, Hainan, Guangxi, Zhejiang, and Yunnan, during 2021 and 2022, including 25 viviparous water lilies and 5 non-viviparous germplasms serving as controls, all of which belong to the tropical water lily genotypes. These seedings were uniformly cultivated at the nursery base of Southwest Forestry University in Kunming, Yunnan Province, China, adhering to standard cultivation practices. Detailed information on Latin names, attribute, sources, and types of all germplasms is provided in Table 6. The materials involved are mostly collected repeatedly in various regions, and are retained based on the growth condition of the plants.

### 4.2. Experimental Method

#### 4.2.1. Investigation of the Morphological Traits

Under identical habitat conditions, and based on the growth status of the plants, a selection of ten plants exhibiting consistent and robust growth was made from each variety in 2023. The period spanning from late July to August marked the peak flowering season, representing an optimal time for observation and appreciation. This timing facilitated a thorough investigation and statistical analysis of morphological characteristics. A total of 34 morphological traits comprising 15 qualitative traits (No. 1~15) and 19 quantitative traits (No. 16~34) were recorded in situ from these 30 germplasms (Table 7), and their inflorescences and mature leaves were measured repeatedly for each trait. This approach ensured comprehensive assessments for morphological characterization. Among them, 21 traits pertained to flower characteristics, including eight qualitative traits (petal and sepal color, petal and sepal spots, anther color, sepal and petal shape, inflorescence form, and flower fragrance), as well as 13 quantitative traits such as length and width of petals and sepals, length of stamen and anther, flower diameter, tepals number, carpels and stamens number, flower height, blooming time of single flower, and flower density. Additionally, there were 12 leaf-related traits including young and mature leaf color, auricle and leaf form, leaf spot, leaf-navel vivipary and diameter, length, width and leaf diameter, auricle length, and epiphyllous bud number, alongside one fruit shape trait.

#### 4.2.2. DNA Extraction and SSR Analysis

During the investigation of morphological traits, fresh young leaves of the water lily were collected, and total DNA was extracted using a modified CTAB method [52]. The quality of the DNA was assessed by 1% agarose gel electrophoresis, and its concentration was measured using a NanoDrop-1000 spectrophotometer (NanoDrop Technologies, Wilmington, NC, USA). High-quality DNA was diluted to 50 ng/μL and stored at −20 °C. Based on the published genome data of *N. ‘Blue Star’*, microsatellite identification (MISA) software was employed to screen SSR loci within the chromosomal sequences of water lilies. In conjunction with previously reported SSR primer sequences for water lilies [20,53]. A total of 16 pairs of SSR primers, characterized by high polymorphism and clear bands, were selected from over 100 pairs of SSR primers. These primers were synthesized by Shanghai Shenggong Biological Engineering Co., Ltd., Shanghai, China.

The PCR reaction system comprised a total volume of 15 μL, containing 7.5 μL of 2 × Taq PCR Master Mix, 2.0 μL of primer mix, 2 μL (50 ng) of DNA template, and 3.5 μL of ddH_2_O. The amplification protocol included an initial denaturation at 96 °C for 3 min, followed by 30 cycles of denaturation at 96 °C for 30 s, annealing at primer-specific temperatures (optimized based on Tm values) for another 30 s, and extension at 72 °C for 1 min, followed by a final extension at 72 °C for 10 min. The amplified products were stored at 4 °C. PCR products were initially detected using 1% agarose gel electrophoresis. High-quality amplicons were further separated by 8% denaturing polyacrylamide gel electrophoresis (PAGE). The PAGE gel was stained in 0.1% silver nitrate solution for 10 min and rinsed briefly for approximately 30 s before being placed in a solution comprising 2% NaOH and containing 0.5% formaldehyde until DNA bands became visible.

### 4.3. Data Processing and Statistical Analysis

#### 4.3.1. Morphological Analysis

Morphological data were collected, organized, and analyzed using Microsoft Excel 2020 software. Qualitative traits were quantitatively coded, and the range and frequency distribution of each trait were calculated. For quantitative traits, we computed statistical parameters including the mean, standard deviation (SD), maximum (Max), minimum (Min), coefficient of variation (CV), and genetic diversity indices. The genetic diversity index (*H’*) was calculated as follows: *H’* = −∑Pi × ln(Pi), where Pi represents the percentage of materials in the i-th of total material, and ln denotes the natural logarithm [54]. Given the heterogeneous measurement scales of morphological traits, Z-score standardization was performed prior to data analysis. Following this standardization process, correlation analysis was performed using Origin 2021 software. Heatmaps were generated at the 0.01 and 0.05 levels [38]. Principal component analysis of morphological traits was performed using SPSS 27.0 software [55]. Principal components were determined based on eigenvalues and cumulative contribution rates, and coefficients of eigenvectors were calculated to establish scoring function equations. An evaluation model for water lily morphological traits was constructed by calculating the F-value that weighted the variance contribution rates of each principal component [56]. Cluster analysis involving 30 water lily germplasms was performed using SPSS 27.0 software based on Euclidean distance metrics [57].

#### 4.3.2. SSR Analysis

According to the manual band scoring criteria, bands amplified by the same primer were evaluated as follows: a clear and smear-free band at an identical position was assigned a score of “1”, while the absence of a band was scored as “0”. This approach established a binary data matrix (0/1) [58]. Data formats were converted with DataFormater software. Genetic diversity indices for SSR loci—including the number of alleles (*Na*), the effective number of alleles (*Ne*), Nei’s gene diversity index (*H*), Shannon’s information index (*I*), the observed heterozygosity (*Ho*), and the expected heterozygosity (*He*)—were calculated with POPGENE 1.32 and GenAIex6.2 [21,59]. The polymorphism information content (*PIC*) values for individual loci were calculated by *PIC* = 1 − ∑P_i_^2^, where P_i_ is the frequency of allele i on the loci [60]. Genetic similarity coefficients (GS) were calculated using NTSYS-PC 2.10e software, followed by principal component analysis (PCA) based on the resulting similarity matrix [61]. Clustering analysis was carried out using the UPGMA (Unweighted Pair Group Method with Arithmetic Mean) within the SAHN (Sequential Aggregative Hierarchical Non-Overlapping) algorithm [62]. The cophenetic values algorithm was applied to convert the clustering results of morphological and molecular markers into cophenetic matrices. Subsequently, the Mx-Comp procedure was employed to evaluate the correlation between the cophenetic matrices and the original similarity coefficient matrices [63]. A Mantel test was conducted to confirm the correlation between the similarity coefficient matrix generated from morphological features and that derived from SSR molecular marker data [64].

## 5. Conclusions

The collection and evaluation of wild water lily germplasms, particularly viviparous germplasms, have been insufficient, and research in this field remains limited. To the best of our knowledge, there have been few studies that investigate the genetic diversity of tropical waterlily germplasms through a combination of morphological descriptors and molecular markers, particularly in relation to viviparous characteristics. The water lily specimens analyzed in this study exhibit distinct morphological variations in leaves and floral organs, along with a relatively high genetic diversity. In addition, to consider the year effect and to ensure the stability of these traits, the results we obtained were all from three-year-old viviparous plants. Nevertheless, the current germplasms collection is limited, and future efforts should focus on expanding the scope and quantity of the viviparous water lily collection, identifying superior genetic germplasms, and initiating timely conservation and breeding programs. These actions will provide high-quality germplasms for the identification, utilization, and development of new water lily germplasms.

## Figures and Tables

**Table 1:** Genetic diversity of 15 qualitative traits in water lily germplasms.

No.	Traits	Frequency Distribution (%)						Genetic Diversity Index (*H’*)
		1	2	3	4	5	6	
1	Leaf auricle	53.33	6.67	40.00	--	--	--	0.888
2	Leaf color	80.00	3.33	16.67	--	--	--	0.585
3	Young leaf color	46.67	26.67	26.67	--	--	--	1.065
4	Leaf form	30.00	30.00	23.33	16.67	--	--	1.366
5	Viviparity of leaf	16.67	83.33	--	--	--	--	0.456
6	Leaf spot	60.00	16.67	23.33	--	--	--	0.950
7	Petal color	10.00	20.00	13.33	33.33	6.67	16.67	1.681
8	Flower fragrance	36.67	40.00	23.33	--	--	--	1.062
9	Petal shape	46.67	30.00	23.33	--	--	--	1.072
10	Inflorescence shape	40.00	60.00	--	--	--	--	0.673
11	Sepal color	20.00	80.00	--	--	--	--	0.500
12	Sepal shape	43.33	6.67	50.00	--	--	--	0.901
13	Sepal and petal spot	30.00	56.67	13.33	--	--	--	0.957
14	Anther color	6.67	26.67	6.67	46.67	13.33	--	1.346
15	Fruit shape	13.33	53.33	33.33	--	--	--	0.969

**Table 2:** Genetic diversity of 19 quantitative traits in water lily germplasms.

No.	Traits	Mean	Max	Min	SD	CV (%)	Genetic Diversity Index (*H’*)
1	Leaf length/cm	18.43	26.19	10.46	4.34	23.54	1.916
2	Leaf width/cm	16.13	21.91	9.35	3.79	23.52	1.972
3	Leaf auricle length/cm	8.23	11.19	4.98	1.96	23.85	1.868
4	Petiole diameter/cm	0.50	0.71	0.29	0.12	23.67	2.007
5	Inflorescence diameter/cm	8.07	11.83	4.60	1.57	19.43	1.532
6	Flower height/cm	9.00	16.46	5.35	3.05	33.84	1.846
7	Single flowering period/d	5.18	7.60	3.50	1.02	19.76	1.949
8	Petal length/cm	4.50	6.29	2.90	0.80	17.73	1.844
9	Petal width/cm	1.21	1.67	0.70	0.23	18.92	1.996
10	Sepal length/cm	19.72	33.30	12.10	4.82	24.43	1.932
11	Sepal width/cm	4.88	6.95	3.00	0.83	16.9	1.963
12	Total number of petals	1.87	2.64	1.10	0.37	19.9	1.775
13	Number of carpels	18.22	22.90	14.40	2.21	12.11	1.913
14	Number of stamens	111.26	164.10	73.60	27.33	24.56	1.762
15	Stamen length/cm	1.83	2.31	1.35	0.25	13.71	1.924
16	Anther length/cm	0.87	1.16	0.53	0.16	18.48	1.950
17	Flower density/20d	4.47	7.93	2.54	1.23	27.51	2.024
18	Diameter of epiphyllous buds/cm	0.56	1.02	0.00	0.33	58.88	1.710
19	Number of epiphyllous buds/cm	2.28	4.60	0.00	1.29	56.56	1.629
Average		--	--	--	2.93	25.12	1.870

**Table 3:** Principal component analysis of 34 morphological traits in water lily germplasms.

Traits	PC1	PC2	PC3	PC4	PC5	PC6	PC7
Leaf auricle	0.117	−0.303	0.198	0.173	0.021	−0.123	−0.182
Leaf color	0.016	−0.079	0.414	0.246	−0.104	−0.040	−0.006
Young leaf color	0.003	0.126	0.360	0.274	−0.064	0.198	0.026
Leaf form	0.045	0.271	−0.153	0.213	0.089	−0.027	0.174
Leaf viviparity	−0.114	0.344	0.166	0.028	−0.083	−0.050	0.207
Leaf spot	0.048	0.032	0.369	0.018	0.061	−0.062	−0.130
Petal color	0.094	0.126	0.220	0.116	−0.117	−0.247	−0.052
Flower fragrance	−0.130	−0.028	0.053	0.136	0.120	−0.478	−0.033
Petal shape	0.087	−0.083	0.153	−0.186	−0.058	0.411	0.144
Inflorescence shape	0.101	−0.218	−0.015	0.056	0.341	0.196	0.137
Sepal color	−0.009	−0.194	−0.020	0.472	−0.123	0.152	0.158
Sepal shape	0.058	−0.084	0.202	−0.190	0.413	0.230	−0.113
Sepal and petal spot	−0.083	0.319	0.143	−0.262	0.041	−0.067	−0.078
Anther color	0.030	0.123	0.044	0.147	−0.026	0.321	−0.526
Fruit shape	0.089	0.037	−0.014	−0.049	0.493	−0.185	0.003
Leaf length/cm	0.299	0.036	−0.057	−0.065	0.074	−0.029	0.141
Leaf width/cm	0.303	0.014	−0.035	−0.066	0.090	−0.036	0.108
Leaf auricle length/cm	0.290	0.093	−0.015	−0.070	0.073	−0.014	0.184
Petiole diameter/cm	0.231	0.053	0.057	−0.226	−0.066	0.174	−0.189
Inflorescence diameter/cm	0.238	0.143	−0.078	−0.048	−0.068	−0.063	−0.199
Flower height/cm	0.208	0.154	−0.128	−0.018	−0.242	−0.102	−0.243
Single flowering period/d	0.068	0.120	0.274	−0.066	0.315	−0.035	0.085
Petal length/cm	0.246	0.104	0.048	0.110	−0.076	0.029	−0.038
Petal width/cm	0.225	0.155	−0.022	0.267	0.142	−0.007	0.073
Total number of petals	0.292	0.129	−0.073	0.063	−0.044	−0.008	−0.002
Sepal length/cm	0.255	0.113	−0.017	0.121	0.001	−0.097	−0.057
Width of sepal/cm	0.172	−0.127	0.270	−0.062	−0.049	−0.122	0.270
Number of carpels	0.223	−0.176	0.098	−0.034	−0.171	−0.094	−0.034
Number of stamens	0.234	−0.185	0.115	−0.056	−0.184	−0.004	0.164
Stamen length/cm	0.280	0.027	−0.147	0.074	0.027	0.082	−0.103
Anther length/cm	0.100	−0.095	−0.273	0.145	−0.044	0.103	0.250
Flower density/20d	−0.047	0.128	−0.117	0.398	0.341	0.068	−0.158
Diameter of epiphyllous buds/cm	−0.006	0.367	0.009	−0.073	−0.080	0.025	0.178
Number of epiphyllous buds	−0.135	0.313	0.120	0.085	−0.053	0.265	0.210
Eigenvalue	9.501	4.238	3.979	2.350	1.829	1.751	1.565
Contribution rate/%	27.943	12.465	11.703	6.911	5.380	5.151	4.603
Cumulative contribution rate/%	27.943	40.408	52.111	59.022	64.403	69.554	74.157

**Table 4:** The ranking of 30 water lily germplasms by calculating related traits.

Germplasm	Principal Component Value							*F*-Value	Ranking
	F_1_	F_2_	F_3_	F_4_	F_5_	F_6_	F_7_		
*N. ‘Purple Joy’*	5.26	3.99	1.49	−2.30	0.42	−0.43	−1.21	2.60	1
*N. ‘Key Largo’*	3.95	1.92	0.27	1.71	0.95	0.57	0.80	2.17	2
*N. ‘Eldorado’*	6.62	−2.06	−1.36	−0.38	0.44	−0.68	−0.02	1.88	3
*N. ‘Carl’s Sunshine’*	1.91	−0.15	3.75	1.96	2.46	−1.46	1.58	1.64	4
*N. ‘Albert Greenberg’*	2.92	−1.15	3.04	0.70	−0.68	1.12	0.80	1.53	5
*N. ‘Blue Bird’*	4.88	−0.82	−1.07	0.44	−1.37	−2.22	0.51	1.35	6
*N. ‘Panama Pacific’*	2.50	2.32	−0.17	−2.27	1.70	−0.25	−0.48	1.17	7
*N. ‘Tropic Sunset’*	1.18	0.66	1.42	1.15	−0.32	−0.58	1.73	0.93	8
*N. ‘Ruby’*	1.82	1.26	−2.17	2.06	0.82	0.99	0.87	0.93	9
*N. ‘Lindsey Wood’*	0.09	2.74	2.06	−2.70	−0.18	1.25	0.02	0.64	10
*N. ‘hybrid’ (red)*	3.65	−3.64	−1.84	−0.22	−2.20	1.03	−1.59	0.27	11
*N. ‘Queen of Siam’*	−0.44	−1.07	3.89	0.03	−0.58	1.23	−0.87	0.26	12
*N. ‘Margaret Mary’*	−0.40	2.07	−1.57	0.34	0.77	2.40	0.46	0.23	13
*N. ‘Ganna Walska’*	−0.31	0.82	2.53	1.72	−2.87	−1.12	−1.64	0.19	14
*N. ‘Micrantha’(blue)*	−1.19	2.26	−0.53	1.36	1.09	−0.98	0.28	0.00	15
*N. ‘George H Pring’*	2.17	−4.71	−1.47	−1.73	0.23	0.98	1.15	−0.21	16
*N. caerulea*	0.04	0.25	−1.13	−0.80	−1.45	−0.64	1.26	−0.27	17
*N. ‘Doris’*	−2.43	−1.15	2.94	1.64	−0.36	0.48	−1.32	−0.57	18
*N. r* *u* *bra*	0.17	−2.24	−1.11	0.43	−0.22	0.27	−2.11	−0.58	19
*N. ‘Black Beauty’*	−2.50	1.18	−0.57	1.51	−1.25	2.06	0.29	−0.62	20
*N. ‘Tina’*	−1.74	0.20	−0.99	0.12	0.09	0.88	1.07	−0.64	21
*N. micrantha (white)*	−1.18	1.32	−1.91	−0.99	−0.71	−2.12	0.83	−0.76	22
*N. ‘Paul Stetson’*	−1.99	−0.15	−1.68	0.62	−0.08	0.16	0.65	−0.94	23
*N. ‘Mameaw’*	−2.71	−0.69	−0.35	−1.52	0.24	1.18	0.76	−1.19	24
*N. ‘Charles Thomas’*	−2.74	−0.66	1.22	−2.88	1.75	−0.43	−2.29	−1.26	25
*N. colorata*	−1.80	−3.25	−1.68	1.91	3.09	−1.01	−2.36	−1.30	26
*N. ‘Candy Rain’*	−3.17	−0.28	−0.85	−2.31	−0.22	−1.16	1.30	−1.61	27
*N. ‘Islamorada’*	−4.38	1.31	−0.29	0.10	−1.47	0.12	−1.02	−1.63	28
*N. ‘Daubeniana’*	−4.11	0.45	−1.59	0.28	−1.71	−2.16	−0.18	−1.98	29
*N. ‘Josephine’*	−6.06	−0.70	−0.29	0.03	1.63	0.52	0.77	−2.24	30

**Table 5:** Genetic diversity analysis of 16 pairs of SSR primers.

Primer	Repeat Motif	Primer Sequence (5′-3′)	T_m_/°C	*Na*	*Ne*	*H*	*I*	*Ho*	*He*	*PIC*
NtG006	(GAA)_5_	F:GGTCTTGCAGACGTCCAAGA R:GATCATCGTCGGCGTCTTCT	58.5	9	7.08	0.43	1.75	0.05	0.45	0.82
NtG011	(AGG)_6_	F:CTCTCATGGCCGACTCATCC R:CGTCTCGTCGACATCAGGAG	58	4	2.01	0.42	1.03	0.12	0.56	0.66
NtG012	(AGG)_5_	F:CGGTTGGGTGAAGATCGGAA R:CGCCGAACTGAAAGACGAAC	59	4	2.11	0.29	1.23	0.23	0.75	0.69
NtG014	(CTC)_7_	F:GGAGACCCAAATGGCCGATT R:CGATCCTTCGTCCTCCGATG	58.5	10	7.48	0.29	1.86	0.32	0.49	0.90
NtG036	(AGA)_5_	F:AGGCCAGAATGCTGTGTTGT R:TGGACATGGATCAGGTGCTT	61	6	4.59	0.20	1.45	0.35	0.75	0.79
NtG042	(CCT)_6_	F:CGCAAAGAGGGAACAATGGC R:CTGTTGCATGCCGGTTATCG	58.5	6	4.65	0.39	1.32	0.22	0.53	0.75
NtG051	(CCA)_6_	F:GAACATGCCTCCACCCATCA R:AGGGAGTTGATGAACAGCGG	58	8	6.88	0.31	1.67	0.08	0.71	0.85
NtG071	(ATG)_8_	F:ATCGCAGATCGGCAGAAGAG R:TTTGCTTGCGTCTCCTCCTT	58.5	7	6.01	0.37	1.87	0.12	0.62	0.79
NtG106	(TCC)_7_	F:AGCAGAACTCAACTCACCGG R:GACCTGCTGGACTTGTCGAA	58	6	5.23	0.36	1.65	0.32	0.76	0.76
NtG107	(CTT)_5_	F:CAGAGACTTACCGCGCTAGG R:GCAGGCAGTTGCGATAGTGA	58	4	2.01	0.47	1.14	0.42	0.57	0.74
NtG111	(ATA)_25_	F:ATTCGAGTGATGGCATGCCT R:AGCCAGCTCGAAGTGACAAA	58	5	3.28	0.27	1.35	0.14	0.73	0.57
NtG115	(GGT)_5_	F:GCAGCAAGTGGTCTCTGTCT R:CTGCTGCTCTGACACCATGA	61	10	7.59	0.26	2.12	0.41	0.81	0.90
NtG146	(TTA)_8_	F:GCCACACCAGCCCACTAAAT R:ATTGAACAGTGGTGGAGCCA	58	7	6.56	0.30	1.85	0.25	0.95	0.75
NtG152	(GTC)_5_	F:GTCCATGTAGTCGTCCAGCC R:GGAAGCGTCGATCAGTGGAT	61.5	9	7.56	0.30	1.87	0.43	0.81	0.85
NtG156	(TCA)_5_	F:ATTTCTCCAACAGCAGGCCA R:CTTACAGGACGTGGGTAGGC	58	9	7.86	0.24	1.95	0.22	0.62	0.80
NtG188	(AGA)_5_	F:ACATGGACGCCAAGCAACTA R:GGAAGCACAAACAGGATGGC	59	10	8.02	0.31	2.25	0.32	0.75	0.86
Mean				7.19	5.56	0.33	1.65	0.25	0.68	0.78

Note: T_m_ is annealing temperature. *Na*, *Ne*, *H*, *I*, *Ho*, *He*, *PIC* represent number of alleles, number of effective alleles, Nei’s gene diversity index, Shannon’s diversity index, observed heterozygosity, expected heterozygosity, and polymorphism information content.

**Table 6:** Relevant information of 30 collected water lily germplasms.

No.	Latin Name of Variety	Attribute	Source	Type
1	*N. ‘Carl’s Sunshine’*	horticultural species	Nanning, Guangxi	V
2	*N. ‘Panama Pacific’*	horticultural species	Nanjing, Jiangsu	V
3	*N. ‘Paul Stetson*	horticultural species	Nanjing, Jiangsu	V
4	*N. ‘Queen of Siam’*	horticultural species	Haikou, Hainan	V
5	*N. ‘Lindsey Wood’*	horticultural species	Nanning, Guangxi	V
6	*N. ‘Mameaw’*	horticultural species	Nanning, Guangxi	V
7	*N. ‘Key Largo’*	horticultural species	Haikou, Hainan	V
8	*N. ‘Josephine’*	horticultural species	Haikou, Hainan	V
9	*N. ‘Purple Joy’*	horticultural species	Haikou, Hainan	V
10	*N. ‘micrantha’ (blue)*	horticultural species	Nanjing, Jiangsu	V
11	*N. ‘Daubeniana’*	horticultural species	Kunming, Yunnan	V
12	*N. ‘Blue Bird’*	horticultural species	Haikou, Hainan	V
13	*N. ‘Tropic Sunset’*	horticultural species	Haikou, Hainan	V
14	*N. ‘Islamorada’*	horticultural species	Kunming, Yunnan	V
15	*N. ‘Doris’*	horticultural species	Haikou, Hainan	V
16	*N. ‘Candy Rain’*	horticultural species	Haikou, Hainan	V
17	*N. ‘Charles Thomas’*	horticultural species	Nanjing, Jiangsu	V
18	*N. ‘Tina’*	horticultural species	Haikou, Hainan	V
19	*N. ‘Ruby’*	horticultural species	Nanjing, Jiangsu	V
20	*N. ‘Black Beauty’*	horticultural species	Haikou, Hainan	V
21	*N. ‘Ganna Walska’*	horticultural species	Haikou, Hainan	V
22	*N. ‘Albert Greenberg’*	horticultural species	Haikou, Hainan	V
23	*N. ‘Margaret Mary’*	horticultural species	Kunming, Yunnan	V
24	*N. caerulea*	initial species	Nanjing, Jiangsu	V
25	*N. micrantha (white)*	initial species	Haikou, Hainan	V
26	*N. colorata*	horticultural species	Nanjing, Jiangsu	NV
27	*N. ‘George H Pring’*	horticultural species	Haikou, Hainan	NV
28	*N. ‘Eldorado’*	horticultural species	Nanning, Guangxi	NV
29	*N. ‘hybrid’ (red)*	horticultural species	Nanning, Guangxi	NV
30	*N. rubra*	horticultural species	Nanjing, Jiangsu	NV

Note: V means viviparous water lilies, NV means non-viviparous water lilies.

**Table 7:** Morphological traits index and standard of water lily germplasms.

No.	Traits	Abbreviation	Criteria for Documenting
1	Leaf auricle	LA	Separated = 1, closed = 2, covered = 3
2	Leaf color	LC	Green = 1, brown = 2, green and brown = 3
3	Young leaf color	YLC	Green = 1, brown = 2, leaves with brown fleck = 3
4	Leaf form	LF	Rotundity = 1, elliptic = 2, oval = 3, broad oval shape = 4
5	Viviparity of leaf	VF	No = 1, yes = 2
6	Leaf spot	LS	No = 1, general = 2, many = 3
7	Petal color	PC	White = 1, amaranth = 2, blue = 3, violet = 4, yellow = 5, compound color = 6
8	Flower fragrance	FF	Strong = 1, refreshing = 2, slight = 3
9	Petal shape	PS	Lance-shaped = 1, oval = 2, spoon-shaped = 3
10	Inflorescence shape	IS	Radial-shaped = 1, cup-shaped = 2
11	Sepal color	SC	Similar = 1, different = 2
12	Sepal shape	SS	Lance-shaped = 1, oval = 2, obovate = 3
13	Sepal and petal spot	SPS	No fleck = 1, fleck of sepal = 2, fleck of sepal and petal = 3
14	Anther color	AC	White = 1, yellow = 2, pink = 3, purple = 4, red = 5
15	Fruit shape	FS	Pineapple-shaped = 1, apple-shaped = 2, peach-shaped = 3
16	Leaf length/cm	LL	Average length per leaf
17	Leaf width/cm	LW	Average width per leaf
18	Leaf auricle length/cm	LAL	Average length of delamination per leaf
19	Petiole diameter/cm	PD	Average diameter of petiole per leaf
20	Inflorescence diameter/cm	ID	Average diameter per flower
21	Flower height/cm	FH	Average height per flower
22	Single flowering period/d	SFP	Average single flower period per flower
23	Petal length/cm	PL	Average length of outer and inner petals per flower
24	Petal width/cm	PW	Average width of outer and inner petals per flower
25	Total number of petals	TPS	Average number of petals per flower
26	Sepal length/cm	SL	Average length of sepals per flower
27	Sepal width/cm	SW	Average width of sepals per flower
28	Number of carpels	CN	Average number of carpels per flower
29	Number of stamens	STN	Average number of stamens per flower
30	Stamen length/cm	STL	Average length of stamens per flower
31	Anther length/cm	AL	Average length of anthers per flower
32	Flower density/20d	FD	Average flower density every 20 days
33	Diameter of epiphyllous buds/cm	EBD	Average diameter of epiphyllous buds per leaf
34	Number of epiphyllous buds/cm	EBN	Average number of epiphyllous buds per flower leaf

## Data Availability

Data are contained within the article.
